# Changes in rainfall distribution promote woody foliage production in the Sahel

**DOI:** 10.1038/s42003-019-0383-9

**Published:** 2019-04-23

**Authors:** Martin Brandt, Pierre Hiernaux, Kjeld Rasmussen, Compton J. Tucker, Jean-Pierre Wigneron, Abdoul Aziz Diouf, Stefanie M. Herrmann, Wenmin Zhang, Laurent Kergoat, Cheikh Mbow, Christin Abel, Yves Auda, Rasmus Fensholt

**Affiliations:** 1 Department of Geosciences and Natural Resource Management, University of Copenhagen, 1350 Copenhagen, Denmark; 2 Pastoralisme Conseil, 30 chemin de Jouanal, 82160 Caylus, France; 3 NASA Goddard Space Flight Center, Mail Code 610.9, Greenbelt, MD 20771 USA; 4 ISPA, INRA Bordeaux, Bordeaux, France; 5 Centre de Suivi Ecologique, BP 15532 Dakar-Fann, Senegal; 6 Agricultural and Biosystems Engineering, The University of Arizona, 1177 E. 4th Street, Tucson, AZ 85721 USA; 7 Geosciences Environnement Toulouse (GET), Observatoire Midi-Pyrénées, UMR 5563 (CNRS/UPS/IRD/CNES), 14 Avenue Edouard Belin, 31400 Toulouse, France; 8 START International Inc., 2000 Florida Ave NW, Washington, DC 20009 USA

**Keywords:** Ecosystem ecology, Climate-change ecology

## Abstract

Dryland ecosystems comprise a balance between woody and herbaceous vegetation. Climate change impacts rainfall timing, which may alter the respective contributions of woody and herbaceous plants on the total vegetation production. Here, we apply 30 years of field-measured woody foliage and herbaceous mass from Senegal and document a faster increase in woody foliage mass (+17 kg ha^−1^ yr^−1^) as compared to herbaceous mass (+3 kg ha^−1^ yr^−1^). Annual rainfall trends were partitioned into core wet-season rains (+0.7 mm yr^-1^), supporting a weak but periodic (5-year cycles) increase in herbaceous mass, and early/late rains (+2.1 mm yr^−1^), explaining the strongly increased woody foliage mass. Satellite observations confirm these findings for the majority of the Sahel, with total herbaceous/woody foliage mass increases by 6%/20%. We conclude that the rainfall recovery in the Sahel does not benefit herbaceous vegetation to the same extent as woody vegetation, presumably favoured by increased early/late rains.

## Abstract (web summary)

Martin Brandt et al. studied foliage mass from Senegal over 30 years, observing the effects of climate change on dryland vegetation. Rainfall caused increases in both herbaceous and woody foliage, but woody vegetation benefits from rainfall timing changes with potential knock-on effects to the local herbivore populations.

## Introduction

Recent Earth observation studies find a greening of the Earth and in particular in global drylands, which is commonly interpreted as a global increase in net primary production and has been attributed to climate change^1,2^. Although changes in rainfall, fire regimes, elevated temperatures, atmospheric CO_2_ and nitrogen depositions are suggested explanations^1,3–6^, only few studies provide quantitative evidence on both the biophysical processes (changes in vegetation cover, structure and composition) and controlling factors of long-term dryland vegetation trends^7^. While Earth observation data have been used extensively to document the spatial and temporal dynamics of vegetation production since the early 1980s^2,8^, satellite observed vegetation dynamics in drylands have rarely been separated into their herbaceous and woody components and validated against field observations^9,10^.

The Sahel dryland was one of the first areas where greening trends were observed^11,12^. Annual rainfall was identified as the major control increasing annual primary production after prolonged droughts in the 1970s and 1980s^13^. However, recent studies show an increased frequency of heavy rainfall events^14^ and a changed seasonal distribution of rainfall^15^ in the Sahel, which may benefit differently the woody and herbaceous components of the vegetation cover. Several experimental field studies in drylands found that woody vegetation benefits from extreme rainfall events^16,17^, and also rainfall falling outside the core of the wet season (early and late rains) is hypothesised to benefit primarily perennial vegetation, which includes woody plants. Herbaceous vegetation in the Sahel are predominantly annual plants wilting towards the end of the rainy season independent of late rains. Yet, the limited availability of continuous long-term field observations of vegetation growth and the failure of satellite systems to readily distinguish between woody and herbaceous vegetation components impedes analysing the link between the dynamics in woody/herbaceous plants and the temporal changes in seasonal rainfall distributions.

The general concept of the so-called ‘greening Sahel’ was based on Earth observation data^12^, yet a few long-term field assessments on vegetation production confirm the general dynamics and trends, including the Gourma region in Mali^11,18^, Fakara in Niger^19^ and the rangelands of Senegal^20,21^. In particular, the arid and semi-arid Ferlo region in northern Senegal has been a testing ground for the use of time series of satellite images since the late 1970s: Field measurements on herbaceous mass were used to evaluate vegetation proxies from NOAA AVHRR satellite data, constituting a pioneer work and creating the backbone of modern research on dryland vegetation monitoring^12–23^. In 1987, the Centre de Suivi Ecologique implemented a routine collection of ecological field data in Senegal, which nowadays represents one of the rare systematic ground surveys using a consistent methodology over more than three decades and measuring both woody and herbaceous vegetation properties. The length of the transects (1 km) forming the surveys was designed for comparisons with Earth observation data and provides robust estimates at a comparable resolution to the dense time series of low-to-medium resolution satellite sensors^23^.

A combination of Earth observation and field data are used here to analyse the contributions of herbaceous and woody plants on long-time vegetation trends, and the impact of specific temporal rainfall distributions on herbaceous and woody plant trends. The convergence of evidence obtained from the combination of field and remotely sensed data highlights the validity of both datasets, as well as the spatio-temporal patterns emerging from them. Furthermore, strong relationships between field and satellite data justify extrapolation of findings from the field plots to larger areas.

Results show that different satellite datasets reliably reflect field-measured dynamics and long-term trends in vegetation production. Whereas optical datasets have difficulties in separating woody and herbaceous vegetation components, vegetation optical depth from passive microwaves is able to assess woody and herbaceous dynamics separately. We find Sahel-wide increases in both herbaceous mass and woody foliage over 30 years, however, woody foliage increases faster than herbaceous mass. This is related to a shift in the timing of rainfall, which benefits primarily woody vegetation. Finally, we find a periodic pattern in the herbaceous production, following a 5-year cycle.

## Results

### Relationship between 30 years of field and satellite data

**Fig. 1:**
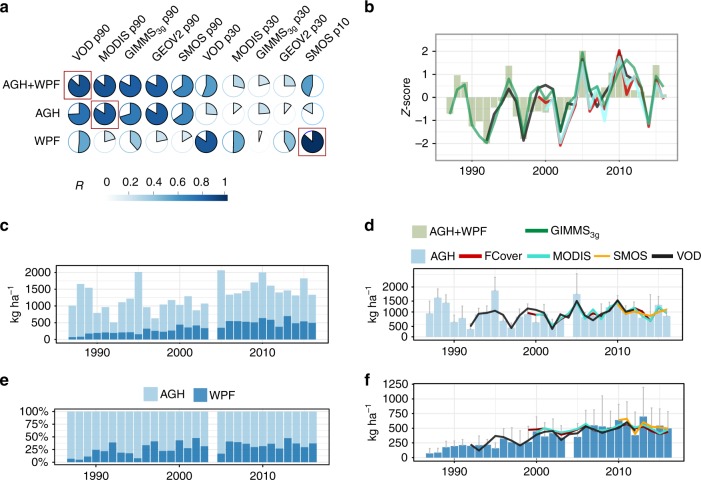
Vegetation dynamics at field sites in the sandy Ferlo (1987–2016). **a** Matrix showing the Pearson correlation coefficient between different satellite datasets (p90 and p30) with total green vegetation mass (AGH + WPF), as well as herbaceous (AGH) and woody foliage (WPF) mass separately (*n* = 30). The highest correlations of each row are marked with a red square. Both the colour and the filling of the circle show the strength of the correlations. P90 is located during the wet season when both woody and herbaceous plants have green leaves, p30 is located in the dry season when only woody plants have green foliage (Supplementary Fig. 2). **b** P90 satellite observations and total green vegetation mass (AGH + WPF). The annual values have been standardised by the mean and standard deviation (*z*-score). Note that the lengths of the time series differ (see Supplementary Table 1). Parts of the legend is shared with (**d**). **c** Field-measured AGH and WPF in kg ha^−1^. **d** Field-measured AGH and p90–p30 satellite observations. **e** The contribution of field-measured AGH and WPF to the total vegetation mass. **f** Field-measured WPF and p30 (p10 for L-VOD) satellite data for Ferlo. All pixels overlaying the nine field sites are averaged per year and satellite product for 1987–2016. Error bars are the standard deviations between the field data plots

We first compared a variety of different Earth observation datasets against field observations on green vegetation mass, consisting of annually collected (1987–2016) above-ground herbaceous mass (AGH) and woody plant foliage mass (WPF) from nine field sites (Fig. 1, Supplementary Fig. 1). The concept of separating herbaceous and woody components using satellite data is based on the contrasting phenology of woody and herbaceous vegetation, with annual herbaceous plants wilting towards the end of the wet season while woody plants keep their green foliage during a fraction of the dry season^24–27^. To reflect the growing season when the annual vegetation mass accumulation is close to its peak (Supplementary Fig. 2), we used the 90th percentile (p90) of intra-annual satellite data time series (averages of all pixels overlaying the nine field sites shown in Supplementary Fig. 1). To avoid bias by specific satellite sensors, we applied a range of different satellite systems having different characteristics as well as spatial and temporal resolutions (Supplementary Table 1). All satellite p90 datasets were able to reproduce total vegetation mass (AGH + WPF; mean 1987–2016 = 1284 kg ha^−1^), as well as AGH trends and dynamics, remarkably well (*r* = 0.69–0.87) with root mean square errors (for WPF + AGH) ranging from 160 kg ha^−1^ (MODIS), 178 (GEOV2), to 215 kg ha^−1^ (GIMMS_3g_) for optical sensors, and 182 and 170 for high- and low-frequency vegetation optical depth (VOD and L-VOD), respectively.

The 30th percentile (p30) of the intra-annual time series was used to represent dry season values estimating WPF (Fig. 1a; Supplementary Fig. 2). Here, performance differences between passive microwave (VOD *r* = 0.85; L-VOD *r* = 0.87) and optical datasets (MODIS *r* = 0.5; GEOV2 *r* = 0.45) were substantial, and the widely used GIMMS_3g_ (*r* = 0.04) was shown to be unsuited to reflect WPF dynamics and is thus not further used in the following sections.

### Herbaceous and woody plant vegetation trends

On average, the portion of field-measured herbaceous mass (AGH) to the total vegetation mass at the field sites was 71% (±13 SD over the nine sites for 1987–2016) (Fig. 1c, f). However, this portion was decreasing over time (–0.7 ± 0.5% yr^−1^) (Fig. 1e), which may reflect the progressive increase in woody populations recovering from the drought years (1983/1984) while the herbaceous mass of a given year is more controlled by rainfall conditions of the current year with less legacy effects from previous years. Indeed, over the period 1987–2016, trends of the field data showed that woody foliage mass (WPF) increases by + 17 ± 13 kg ha^−1^ yr^−1^, which is considerably more than AGH (+3 ± 8 kg ha^−1^ yr^−1^). However, large inter-annual variations make a linear trend estimate on herbaceous mass largely dependent on the selection of the start and end years of the time series, which is not the case for woody foliage (Supplementary Fig. 3; Fig. 1b, c). Satellite data confirm the patterns observed in the field data (Fig. 1b, d, f).

**Fig. 2:**
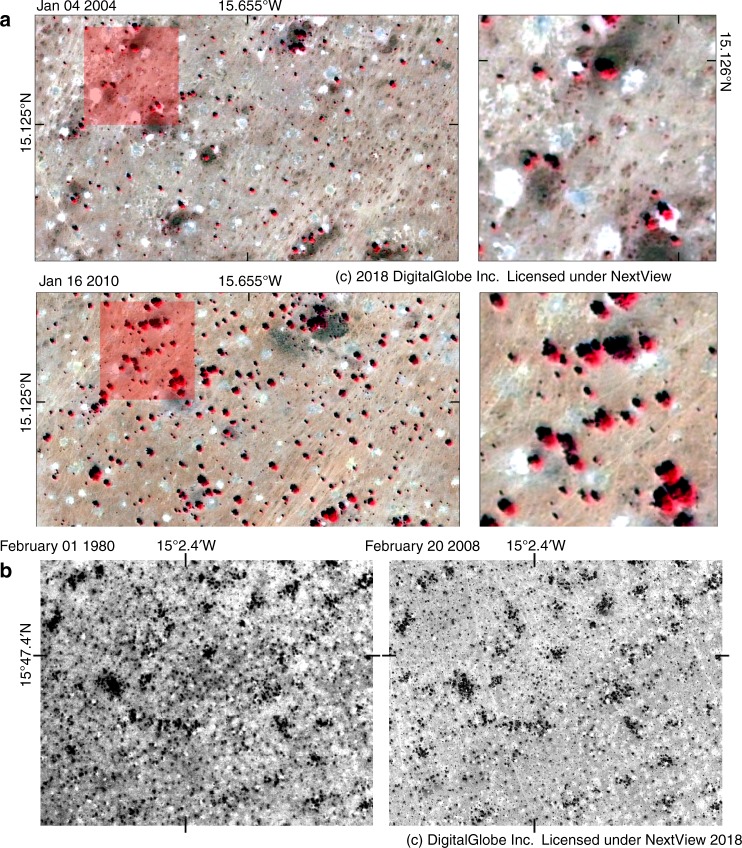
Illustration of woody population dynamics. **a** Image pair of Quickbird-2 images from January 2004 and 2010 showing a burst of growth in both canopy size and tree density in the southern part of the sandy Ferlo. Images are shown in false colour composites (bands 4,3,2) with trees as red objects. Bright white dots are eroded ancient termite mounts without any vegetative cover. Field-measured woody cover was 2% in 2005 and 6% in 2009. WPF was 331 kg ha^−1^ in 2003 and 468 kg ha^−1^ in 2010. Additional image pairs can be found in Supplementary Figs 4–6. **b** This image pair (aerial photo from 1980 and WorldView-1 from 2008) shows no obvious changes in woody cover over several decades. Optical differences are mainly caused by the different observation systems

Although the general pattern seen in Fig. 1 is clear, spatial discrepancies inevitably exist, and WPF can vary locally between the years. Further insights were provided by independent qualitative datasets: very high resolution imageries illustrate a recent burst of growth in both tree canopy size and density over 6–11 years (Fig. 2a, Supplementary Fig. 4). However, a visual comparison of aerial photos from 1980 covering most of the study area with imagery from 2008 showed that the increase in WPF was not obviously associated with a widespread encroachment of individual woody plant density (Fig. 2b, Supplementary Figs 4–6). This shows, first, increases in WPF are not necessarily coupled with an increased plant density, second, increases in woody cover are not always homogeneous but site dependent, and third, the increases in WPF are likely linked with the recovery from the drought in 1984 which had caused a mass dying of woody plants, which today are back at pre-drought level (as shown in 1980).

### Herbaceous and woody vegetation respond to rainfall patterns

**Fig. 3:**
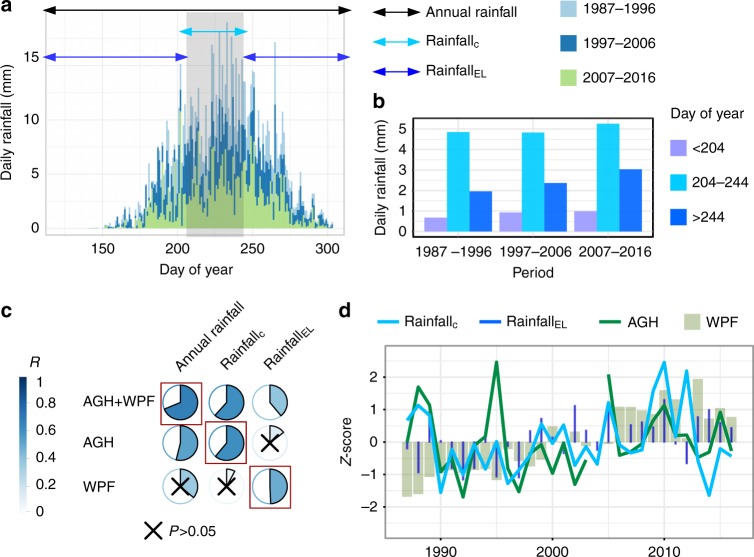
Rainfall distribution impact on vegetation composition in the Ferlo. **a** Daily rainfall estimates overlaying the nine field sites averaged for three 10 year periods. The arrows mark the averaged (1987–2016) start and end dates: core wet-season rains (rainfall_C_; cyan arrow and grey shaded box) is useful for annual herbaceous plants and falls on average between day of year 204 and 244 following a ruleset applied to the field sites. The remaining early and late rains (rainfall_EL_) are shown as dark blue arrows. Both components sum up to annual rainfall (1st May to 31st October). **b** Mean daily rainfall for rains before day of year 204 (left bar), 204–244 (middle bar), and after day of year 244 (right bar) averaged for three periods. **c** Field-measured herbaceous (AGH) and woody foliage (WPF) mass are correlated with annually decomposed CHIRPS based rainfall metrics (1987–2016). Crosses mark insignificant (*P* > 0.05) correlations. **d** Temporal dynamics in field-measured herbaceous (AGH) and woody foliage (WPF), as well as annually decomposed rainfall components illustrated in (**a**) (*z*-scores)

Since annual herbaceous plant growth is primarily sensitive to rainfall during the core wet season^24^, we decomposed the annual rainfall (from 1st May to 31st October excluding the core dry season) into rainfall_C_ that is uninterrupted core wet season rainfall, and rainfall_EL_, which is early and late rains falling before and after the rainfall_C_ period (Fig. 3a, b). The decomposition is based on a ruleset adapted from Hiernaux and Le Houérou^28^ (details provided in Methods section). On average across the nine field sites and 30 years, the core wet season lasts from day of year 204 to 244. Over the 30-year study period, rainfall during the average core wet season remained rather stable, while rainfall both before and after this period increased (Fig. 3a, b). Annual rainfall was highly related to field-measured AGH + WPF (*r* = 0.7; *P* < 0.0001), confirming the annual rainfall amount as the major driver of annual vegetation production but also leaving room for other explanatory variables (e.g. soil, herbivores, runoff, etc.) (Fig. 3c). AGH was significantly related to annual rainfall (*r* = 0.54; *P* = 0.002), which was not the case for WPF (*r* = 0.36; *P* = 0.06). Annually decomposed rainfall_C_ showed a stronger relationship with AGH as compared with annual rainfall (*r* = 0.61; *P* = 0.0004) (Fig. 3c, d) but was not significantly related to WPF (*r* = 0.08; *P* = 0.68). Indeed, the only rainfall variable showing a significant correlation with WPF was the annually decomposed rainfall_EL_ (*r* = 0.57; *P* = 0.0006), which was unrelated to AGH (*r* = 0.13; *P* = 0.45).

**Fig. 4:**
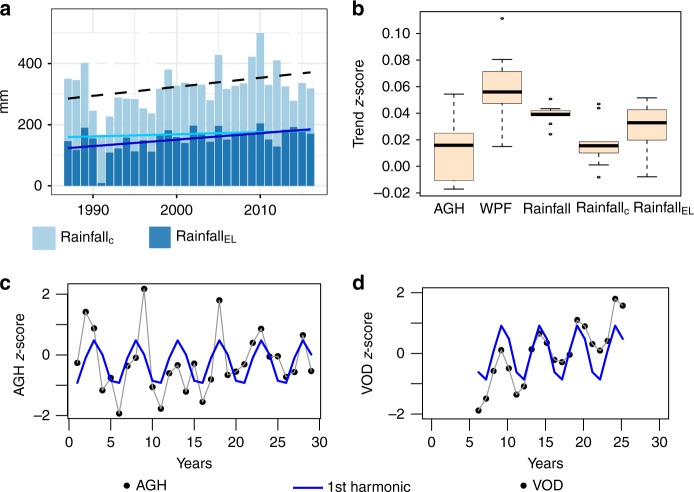
Rainfall patterns in Ferlo 1987–2016. **a** Annual rainfall (trend line back and dashed) is decomposed into core wet-season rainfall (rainfall_C_; light blue trend line) and early and late rains (rainfall_EL_; dark blue trend line.) **b** Temporal trends in the standardised (*z*-score) variables: above-ground herbaceous (AGH) and woody foliage (WPF), as well as core wet season (rainfall_C_) and early/late rains (rainfall_EL_). **c** Annual herbaceous vegetation production (black dots connected by grey lines) follows a periodic pattern shown as a fitted sinusoidal term (1st harmonic from a Fourier transformation) which is also seen in (**d**), the wet-season values of VOD (p90), shown as black dots connected by grey lines. Analyses of more long-term datasets are shown in Supplementary Fig. 7. All analyses are based on the nine field sites

The positive trend seen in annual rainfall (+3 mm yr^−1^; 1987–2016) was less pronounced in rainfall_C_ (+0.7 mm yr^−1^), whereas rainfall_EL_ increased by +2.1 mm yr^−1^ (Fig. 4a, b), suggesting that the general increase found in annual rainfall is caused by an increase in early and late rains (Fig. 4a). This implies that the ratio between rainfall_C_ and rainfall_EL_ changed to the detriment of rainfall_C_ over the 1987–2016 period (–0.4% yr^−1^). The standardized trend seen in rainfall_C_ is comparable to the standardized trend in AGH (Fig. 4b) whereas the magnitude of the standardized trend of rainfall_EL_ approaches the standardized trend seen in WPF (Fig. 4b). These results support the assumption that woody vegetation, unlike herbaceous vegetation, prospers from early and late rains that fall outside of the core of the wet season during which the main growth of herbaceous plants occurs.

Although the general long-term changes in AGH and WPF follow a linear trend, the overall significance of AGH trends is low and the deviations from a linear increase appear to follow a dominating periodicity (Fig. 1b and Fig. 4c, d, Supplementary Fig. 7). To test for recurrent patterns, we fitted a sinusoidal term (1st harmonic) on AGH and the annual VOD p90 time series for the Ferlo (Fig. 4c, d). We found a clear and significant periodicity in both AGH (*P* = 0.009) and in VOD (*P* = 0.003), with a ~5-year cycle length following the 1st sinusoidal term. This periodic pattern was less clear in annual rainfall (*P* = 0.08) but was found to be significant for rainfall_C_ (*P* = 0.03) and not signifcant for rainfall_EL_ (*P* = 0.09). The periodic 5-year cycle was also not present in the field-measured WPF (*P* = 0.7).

### Scaling field-site observations to the Sahel

Based on the strong linear correlation between VOD metrics and both AGH and WPF field data from the Ferlo, we used VOD to test whether the three major patterns observed at the field site level apply also for the western and central Sahel (150–600 mm rainfall per year, derived from mean CHIRPS 1981–2016): first, the stronger increase in WPF as compared with AGH, second, stronger increase in early/late rain as compared with core wet-season rain, and third, the periodic cycles in AGH superimposing the long-term positive trend.

**Fig. 5:**
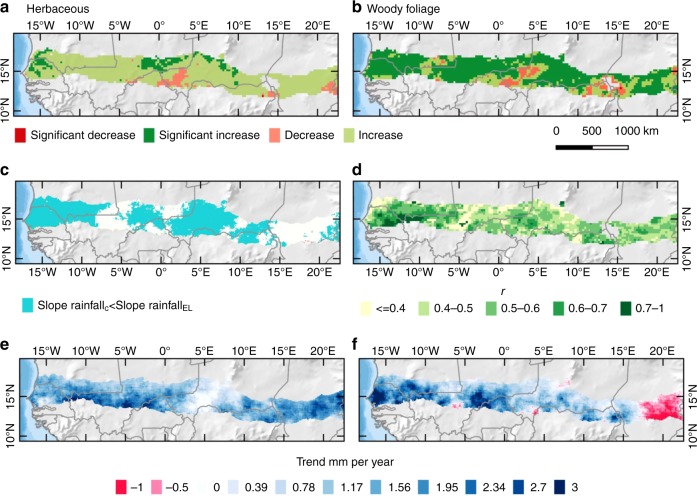
Trends for the Sahel (1992–2012). **a** VOD estimated herbaceous mass (AGH) trends. **b** VOD estimated woody foliage (WPF) trends. Significant (*p* < 0.05) trends are shown in dark green and red, insignificant trends (*p* > 0.05) are shown in light red and green. **c** Areas where the slope in rainfall_EL_ (early and late rains) is larger than the slope in rainfall_C_ (core wet-season rains) are shown in blue colour. **d** Pearson correlation between annual VOD p90 and pixel-wise fitted sinusoidal term (1st harmonic). **e** Core wet-season rainfall (rainfall_C_) trend. **f** Early and late (rainfall_EL_) trend

**Fig. 6:**
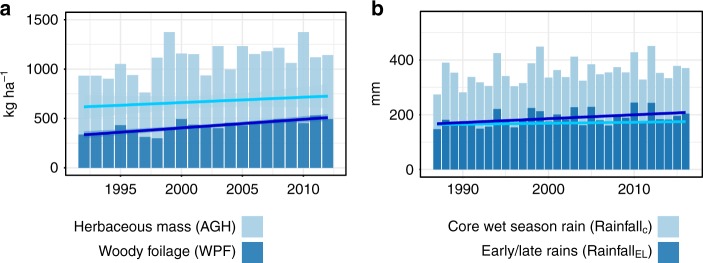
Trends in vegetation composition and rainfall distribution in the western Sahel. VOD-based estimates of herbaceous mass (AGH; light blue) and woody foliage (WPF; dark blue) for the Sahel (1992–2012) as delineated in Fig. 5. Linear trends are shown as light and dark blue lines. The slopes for AGH (5 kg ha yr^−1^) and WPF (9 kg ha yr^−1^) are significantly different (*P* < 0.01). **b** Decomposed rainfall trends for areas with a higher slope in early/late rains (rainfall_EL_) than core wet-season rains (rainfall_C_) (*n* = 35 704) for 1987–2016 (*n* = 30). Rainfall_C_ corresponds to the core wet-season rain and rainfall_EL_ to the early and late rains. The slopes for rainfall_C_ (0.4 mm yr^−1^) and rainfall_EL_ (1.4 mm yr^−1^) are significantly different (*P* = 0.01)

Firstly, we analysed long-term herbaceous and woody vegetation dynamics (Fig. 5a, b, Fig. 6a). According to fitted trends derived from VOD metrics from 1992 to 2012, AGH increased by + 5 ± 7 kg ha^−1^ yr^−1^ and WPF increased by +9 ± 8 kg ha^−1^ yr^−1^. For the entire western and central Sahel area, the summed AGH increased by 0.0025 Pg yr^−1^ over the 21-year period, and WPF by 0.004 Pg yr^−1^. Total AGH (WPF) was 0.28 Pg (0.16 Pg) in 1992 and 0.31 Pg (0.24 Pg) in 2012, which is a relative increase of 6% for AGH and 20% for WPF. The share of VOD estimated WPF (as related to the total vegetation mass) increased from 35% in 1992 to 43% in 2012, which is comparable to the percentage numbers observed from the Ferlo field samples. Areas with a significant (*P* < 0.05) increase in AGH cover a smaller region (289,375 km²; ~7%) than areas with a significant increase in WPF (2,711,875 km²; ~64%) with decreases in vegetation production being mostly not significant (Fig. 5a, b). Significant (*P* < 0.05) increases in AGH were mostly located in the Ferlo (Senegal) and the northern Sahel of Mali, which is in line with field observations shown in ref. ^11^. Areas with a significant increase in WPF are observed across the entire Sahel; notably in Senegal, Chad, eastern Niger and large parts of Mali. Inter-annual variations were lower for WPF (SD 60 kg ha^−1^) than for AGH (SD 137 kg ha^−1^). Decreases in both AGH and WPF were observed in western Niger and around Lake Chad, with no obvious relationship with rainfall, pointing towards other, rainfall independent causes (for example land management).

Secondly, when averaged over the western and central Sahel for 1992–2012, rainfall_C_ increases by +0.9 mm yr^−1^ and rainfall_EL_ by +1.4 mm yr^−1^ (Fig. 5c, e, f, Fig. 6b). In 60% (68% for 1987–2016) of the western Sahel, rainfall_EL_ increases more than rainfall_C_, here the slopes were +0.7 mm yr^−1^ for rainfall_C_ and +1.8 mm yr^−1^ for rainfall_EL_. Only in Chad the opposite was the case (Fig. 5c), suggesting other reasons than rainfall distribution as driver of the observed WPF increase.

Thirdly, the correlation between annual VOD p90 and the per-pixel fitted sinusoidal term was predominantly high over most areas, except for wetlands and some sparsely vegetated areas (Fig. 5d). Higher correlation values are found in the western part of the study area, in proximity to the Atlantic Ocean.

## Discussion

Our study confirms the greening Sahel as an increase in vegetation production, and the convergence of independent field and satellite datasets leaves little room for data uncertainties (calibration procedures, atmospheric correction, orbital drift, and so on) impacting on the trend^11^.

Using field and satellite data, we were further able to disentangle the trend and dynamics in herbaceous and woody plant foliage mass components. Here, passive microwave systems confirmed the convincing results from earlier studies using VOD to estimate woody cover dynamics^3^. An advantage of VOD data over optical satellite systems is the ability to overcome the shortcoming related to the weak signal of woody plants in areas with a woody cover below 10%, where optical satellite images are prone to noise and soil reflectances impacted by straw and litter^26^. L-VOD data, on the other hand, showed an excellent agreement with field-measured WPF, even though data from the low-frequency sensor were only available for an 8-year period. Our results show the importance of L-VOD data for monitoring vegetation biomass in a semi-arid monsoonal ecosystem, as it is operational at an almost daily basis and insensitive to contamination by clouds and atmospheric perturbations, enabling a regional scale assessment of available vegetation mass already during the cloud-prone rainy season. Moreover, whereas the assessment of herbaceous mass by satellite data has long been established at a high quality^29^, the possibility to directly assess woody foliage mass is a milestone in dryland research.

We found increases in both herbaceous and woody foliage mass during the period of analysis; however, woody foliage mass increased faster than herbaceous mass. These trends were found to be closely related to changes in the seasonal distribution of rainfall as derived from daily satellite-based rainfall estimates. The increase in Sahelian annual rainfall was more driven by early and late rains that are less utilised by annual herbaceous plants, explaining the moderate increase in herbaceous mass. Annual herbaceous vegetation needs a period of concentrated rainfall (core wet season) to germinate, tiller, head, flower and set seeds, and they wilt towards the end of the wet season, independent of late rains (photoperiodicity)^24,27^. Moreover, annual herbaceous plants are vulnerable to dry spells, given their shallow rooting depth^30^. Contrastingly, woody plants are able to make use of early and late rains^17^, mostly explaining the rapid increase in woody foliage mass under the tendency of Sahelian rainfall increases shifting to the early and late season. These findings are critical and would imply that the traditionally strong linkage between annual rainfall and growing season net primary production could be weakened^10,31,32^. Moreover, recent findings showing an intensification of Sahelian rainfall^13^, including an increased frequency of large rainfall events^33^, likely contribute to the observed phenomenon. Indeed, large rainfall events are only partially used by annual plant due to rainfall runoff/on, and thus woody vegetation, which is often spatially concentrated in depressions^16,18^, will prosper from more water redistribution after large rainfall events.

The observed trends in herbaceous mass was, however, not linear, and follow a periodic pattern rather than a linear trend, with a recurrent cycle of ~5 years. This pattern is also found in the satellite data and follows a sinusoidal term, which was less pronounced in the total annual rainfall, but significant (*P* < 0.05) for core wet-season rainfall. The potential mechanisms behind this recurrent cycle of years with high and low herbaceous vegetation production and core wet-season rainfall were not further explored in this study. However, it indicates a relation to the forcing from sea surface temperature (SST) anomalies on vegetation growth in the Sahel via the SST impact on rainfall^34^. The reasons for the large inter-annual and decadal fluctuations in rainfall are still not entirely understood, but a study by Sheen et al. ^35^ predicted wet season rainfall variability at both multi-year and inter-annual time-scale driven by the interplay between north Atlantic and Mediterranean SSTs (associated with multi-year (<5 year) cycles) and the El Nino-Southern Oscillation (ENSO) (primarily associated with inter-annual rainfall variability). These driving mechanisms help explaining the periodicity we observed in herbaceous vegetation growth, which is largely controlled by rainfall^31^.

The CMIP5 modelling experiment, reported in IPCC AR5, represents the main source of information on expected future climate trends for the Sahel. However, differences between rainfall projections of the models included are large, while an increase in atmospheric greenhouse gases and temperature is certain. There are indications that westernmost Sahel may experience a reduction in rainfall, while trends are insignificant (*P* > 0.05)—or slightly positive—further eastward. If an increase in occurrence of heavy rainfall as well as early and late rainfall events continue in the Sahel, a likely consequence will be that woody vegetation will benefit, involving an increase in woody cover in low-lying run-on areas, as already observed in parts of northern Burkina Faso^36^. Finally, our study did not explore drivers other than rainfall, and it is possible that elevated atmospheric CO_2_^37–39^ and a declining fire frequency^6,8^ play a role in the increased woody foliage production. Another possible driver is grazing pressure^8,38^, however, for the Sahel, there is no clear relationship between grazing pressure and woody vegetation cover^40^.

The observed increase in vegetation mass has implications for pastoralists and agro-pastoralists, as increasing amounts of fodder mass are becoming available than in the 1980s and 1990s. However, a considerable portion of the observed increase in vegetation production consists of woody foliage mass, the fodder value of which is generally lower than that of herbaceous plants and would benefit primarily browsers (goats) over grazers (sheep and cattle).

Yet, it remains a challenge to directly relate the increase in woody foliage mass production with woody plant density and the percentage coverage. Actual changes in percentage of woody cover (number of woody plants and canopy size) are generally subtle in the Sahel^26^, in contrast to observations from Southern Africa^37–39^, where a massive encroachment of thorny woody plants represents a problem for exploitation of ecosystem services for livestock farmers^38^. Indeed, an increase in production does not necessarily coincide with an expansion in plant density, and does also not exclude the replacement of trees by shrubs^41,42^. Although very high spatial resolution imagery gave evidence on an increased density of trees at some places in recent years, and also field data starting in 2000 showed a considerable increase in woody cover (Supplementary Fig. 8), this cannot be generalised to the Sahelian scale for the period of study, as an increased production can also be caused by increased leaf density or crown area, with no considerable change in plant density. To relate the increased woody production with tree and shrub density, species, canopy sizes and leaf density, more studies at the level of individual trees are needed, which at large spatial scales is only viable from the use of large quantities of very high spatial resolution satellite data, currently being cost-prohibited. Finally, our analysis starts approximately a decade after the great droughts and it is possible that the woody cover prior to the droughts in the 1970s and beginning 1980s was generally higher than what we observe nowadays^42^. Nevertheless, the strong relationship between increasing early and late rains (rainfall_EL_) and increasing leaf production of woody plants (WPF) implies that the recovery of rainfall after the Sahel droughts only partly benefits herbaceous plants being the prime fodder resource of the Sahel. It has yet to be tested how these findings relate to greening trends and altered rainfall conditions in global drylands.

## Methods

### Concept

This study aimed at deriving simple and reproducible metrics of vegetation composition (herbaceous and woody) and rainfall from satellite data to use these to establish relationships with field-measured vegetation production, separated in woody plant foliage mass (WPF) and above-ground herbaceous mass (AGH), and to study inter-annual dynamics in woody and herbaceous production and rainfall over three decades. Field data were obtained for the Ferlo in northern Senegal (Supplementary Fig. 1), and results are evaluated over the Sahel (150–600 mm annual rainfall). We applied state-of-the art optical and passive microwave satellite data, as well as very high spatial resolution imagery from commercial satellites. For each year and time series data set, we applied the 90th percentile (p90) of the observations as proxy for the growing season values. The 30th percentile (p30) was used to assess dry season values. We tested different variables representing the wet and dry season (for example seasonal maximum and minimum) with comparable results but lower correlations with the field data; the stability of the metrics is illustrated in Supplementary Fig. 2. Daily satellite-based rainfall estimates were used to decompose the annual rainfall in core wet-season rainfall (rainfall_C_) on the one hand, and early and late rains (rainfall_EL_) on the other hand. These rainfall metrics were analysed together with vegetation production measurements.

### Field data and study area

Field data was obtained from the Centre de Suivi Ecologique database and included annual measurements of nine field sites from the sandy Ferlo in northern Senegal, each covering a 1 km transect and located in rangelands selected to be distant from livestock concentration spots. At each site, two components were measured annually from 1987 to 2016: AGH (kg ha^−1^) and WPF (kg ha^−1^). The methods are described in detail in refs. ^23,29^. The nine field sites are representative for the sandy Ferlo, a pastoral region with sandy soils in northern Senegal with a mean annual rainfall of 328 mm (from 1982 to 2016). Woody vegetation is generally scattered and the crown cover fraction is below 10% (with a mean WPF of 362 kg ha^−1^), but trees can be concentrated and woody cover reaches 40% in more loamy inter-dune depressions^43^. Herbaceous vegetation consists of annual plants (mean AGH = 939 kg ha^−1^) which wilt towards the end of the rainy season turning into straw and litter^27^. The mass of straws and litter decreases progressively during the dry season due to livestock grazing and trampling, insect herbivory and organic decomposition. The sum of AGH and WPF reflects the total green vegetation mass. The homogeneous landscapes of the Ferlo (homogeneous here referred to as a landscape characterized by similar spatial texture at scales ranging from 100’s to 1000’s of metres) make it well-suited for establishing relationships with medium to coarse resolution satellite data^44^.

### Satellite data

Satellite time-series data covering several decades are becoming increasingly available and new generations of data processing technologies have considerably improved vegetation monitoring by offering independent and complementary information. Satellite data applied in this study can be grouped into optical indices derived from AVHRR sensors (GIMMS_3g_ v1 NDVI; 1982–2016), SPOT VGT and PROBA-V (GEOV2 v2.02 FCover; 1999–2016) and MODIS (MOD13C2 collection 6 NDVI; 2000–2016) and VOD indices derived from passive microwave satellite systems. One VOD index used here is based on recordings in the high wavelength frequency domain (termed VOD hereafter; 1992–2012) and another is based on SMOS observations; the first passive microwave radiometer operating at a low frequency (L-band, hence termed L-VOD; 2010–2016). Two of the time series can be considered as long-term (GIMMS_3g_ and VOD), whereas the remaining datasets only start in 1999 or later. Apart from SMOS L-VOD, we selected satellite datasets that were already pre-processed, including the selection of the best-quality observations, cloud filtering, gap filling and temporal smoothing. Also, adjustments to the use of varying sensors had already been performed and we refer to the respective product guides for further information^45–48^. For L-VOD, we used the SMOS-IC version and filtered out low-quality observations following^49^ and aggregated daily images to 10-day medians which were further filtered with a local weighted regression (2nd order polynomial fit). The temporal resolution of the datasets ranged from 10 days (GEOV2, SMOS-IC), 15 days (GIMMS_3g_) to 1 month (MODIS, VOD).

Very high spatial resolution imagery from Worldview-2, Quickbird-2 and GeoEye-1 commercial satellites were included only for visual illustration purposes^43^. The very high spatial resolution images were all pansharpened to a 50 cm resolution.

### Rainfall

We used daily CHIRPS v2.0 gridded rainfall estimates which blend station and satellite-based rainfall data at 5.6 km spatial resolution^50^. To study the impact of rainfall distribution on herbaceous and woody vegetation growth, we separated the cumulative annual rainfall into two parts: the rainfall during the core of the wet season (rainfall_C_) which is the annual amount of rainfall that can be utilised by annual herbaceous plants, including rainfed crops. It corresponds to a temporal window during the wet season which is not interrupted by dry spells. Rainfall_EL_ (early and late rains), which is the annual amount of rainfall falling between 1st May and 31st October and before or after the core wet-season rains (Fig. 4a). The core dry season (November, December, January, February, March, April) was excluded due to data uncertainties in the rainfall data^15^ and the possibility of herbaceous vegetation flushes caused by out-of-season rains. The calculation of rainfall_C_ and rainfall_EL_ is based on the following steps and definitions^28^:

To simulate potential evapotranspiration, two daily time series of cumulative rainfall (CR) are established: CR-5 accumulates daily rainfall subtracting 5 mm each day (when available), CR-3 accumulates daily rainfall subtracting 3 mm each day (when available). CR-5 and CR-3 cannot be negative (negative values are set to 0).

Four dates are to be established to separate core wet-season rains (rainfall_C_) from early and late rains (rainfall_EL_):

*Onset first rain*: Either the first day with at least 12 mm daily rainfall or first day of five consecutive days with a sum of >20 mm. *Onset core wet season*: Either 10 days after the onset of the first rain with CR-5 being positive if no dry spells occur (these days are necessary for the germination), or first day with CR-5 positive after the last dry spell, but before 15th August. *End of core wet season*: After 15th August, and before possible dry spells, i.e. if a dry spell occurs between 15th August and the last rain, the core wet season ends. *Last rain*: After 15th August, the last day with rainfall above 12 mm or five consecutive days with a sum of >20 mm.

Dry spells are identified if any of the following conditions are true:

Six consecutive days where CR-3 is zero. Twelve consecutive days with summed CR-3 balance below 6 mm. Eighteen consecutive days with summed CR3 balance below 9 mm. Twenty-four consecutive days with summed CR3 balance below 12 mm. Thirty consecutive days with summed CR3 balance below 15 mm.

If two or more of the conditions apply, the longest dry spell prevails. The dates were identified for the CHIRPS pixels overlaying the nine field sites and the start and ending date of the core wet season were derived for each year and site (1987–2016). The rainfall summed over this period was termed rainfall_C_, and subtracted from the annual rainfall (that is from 1st May to 31st October) to derive rainfall_EL_. The average starting date of rainfall_C_ was day of year 204 and the average ending date was day of year 244. These two dates were used to establish the core wet season for the analysis at Sahel scale, without considering the latitudinal rainfall gradient. Using these two approaches of various complexities (one which takes each year’s rainfall distribution into account when assessing start and end dates and a simpler approach using fixed dates) also gives evidence on the robustness and replicability of the results.

From literature, perennial plants (including woody vegetation) are not limited to a strict rainfall pattern^27,51^. Nevertheless, the establishment of a clear statistical relationship between rainfall and plant growth is difficult, since runoff/run-on, topography, herbivores, species composition and soil fertility are factors impacting on the relationship but are not taken into account in this study.

### Calibration of satellite data to vegetation production

To study inter-annual dynamics in vegetation production with both satellite and field data, we used the nine field sites and averaged p90 and p30 of each satellite data set for each year overlaying the sites. It is expected that using an average over several sites reduces noise introduced by the different spatial resolution of the datasets^52^, which varied from 1 km (field data, GEOV2), 5.6 km (MODIS), 8 km (GIMMS_3g_) to 25 km (VOD and L-VOD). Due to the varying spatial resolutions, it cannot be avoided that different spatial areas are inter-correlated, but this does not affect the temporal dynamics^52^. The application of p30 does not fit the characteristics of the SMOS sensor (the low frequency is less sensitive to small vegetation components, i.e herbaceous vegetation, leading to a very low seasonality^53^), so instead we applied p10 in the case of SMOS to capture the low values of the dry season (p30 of SMOS would be located in the wet season).

To estimate annual vegetation production with satellite data, the coefficients of a linear regression between annual satellite metrics and field data (average of 9 sites) were applied to the annual satellite imagery. P90 (reflecting the growing season) was used to estimate the total vegetation mass, and p30 (reflecting the dry season) for the WPF. The availability of in situ observations that separate AGH and WPF provide an opportunity to assess the validity of satellite-derived WPF dynamics. Derivation of WPF from satellite data is based on the contrasting phenology of woody and herbaceous vegetation, with annual herbaceous plants wilting at the end of the wet season while woody plants keep their green foliage during a fraction of the dry season. Dry season satellite imagery can thus be used to capture the WPF component separately from the AGH component^26^.

1 $$\left( {{\mathrm{AGH}} + {\mathrm{WPF}}} \right) = {\mathrm{VOD}}_{{\mathrm{P}}90} \ast {\mathrm{slope}} + {\mathrm{offset}}$$

2 $${\mathrm{WPF}} = {\mathrm{VOD}}_{{\mathrm{P}}30} \ast {\mathrm{slope}} + {\mathrm{offset}}$$

3 $${\mathrm{AGH}} = \left( {{\mathrm{AGH}} + {\mathrm{WPF}}} \right)-{\mathrm{WPF}}$$

Assuming that WPF is present at both dry and wet season whereas AGH is only present during the wet season, AGH was estimated by subtracting WPF (estimated by p30) from AGH + WPF (estimated by p90). Equations 1–3 exemplify the calculations for the VOD data set (assuming that the correlation between VOD and field data is strong). It should be noted that the relationship was established with a spatial average at an annual scale, mainly addressing inter-annual variations but not the spatial heterogeneity, which is addressed elsewhere^23^. The calibration between satellite and field data was done over the Senegalese Ferlo, and the coefficients were subsequently applied over the entire West African Sahel. This is deemed appropriate as the entire Sahel zone shares similar conditions: a monsoonal climate with rainfall only from June to October, predominant pastoral use, scattered woody vegetation with an average coverage of 3% (150–300 mm rainfall) and 9% (300–600 mm rainfall) dominated by evergreen species^42^, and annual herbaceous vegetation. Since our analysis does not exceed 600 mm annual rainfall, perennial herbaceous plants are not common.

### Reporting Summary

Further information on experimental design is available in the Nature Research Reporting Summary linked to this article.

## Supplementary information

Reporting Summary

Supplementary information

Description of additional supplementary items

Supplementary Data

## Data Availability

CHIRPS rainfall data is freely available at the Climate Hazard Group (http://chg.geog.ucsb.edu/data/chirps/). SMOS and L-VOD data are available via CATDS (Centre Aval de Traitement des Données SMOS) at https://www.catds.fr/. GEOV2 data are kindly provided by the Copernicus Global Land Service (http://land.copernicus.eu/global/). VOD data were provided by Yi Liu (available at http://www.wenfo.org/wald/global-biomass/). The data used for this study are available in text format in the supplementary part (Supplementary Data). The copyright for the field data remains at the CSE, Senegal. Commercial very high-resolution satellite images were acquired within the NextView license program. The copyright remains at DigitalGlobe and a redistribution is not possible.
