# Supplementary material for: Changes in rainfall distribution promote woody foliage production in the Sahel
Source: Commun Biol. 2019 Apr 23;2:133. doi: 10.1038/s42003-019-0383-9 (PMC6478729; doi:10.1038/s42003-019-0383-9)
Supplement: Supplementary file 1 — Reporting Summary [file 42003_2019_383_MOESM1_ESM.pdf]

nature research

Corresponding author(s): Martin Brandt

Last updated by author(s): Nov 27, 2018

Reporting Summary

Nature Research wishes to improve the reproducibility of the work that we publish. This form provides structure for consistency and transparency in reporting. For further information on Nature Research policies, see Authors & Referees and the Editorial Policy Checklist.

Statistics

For all statistical analyses, confirm that the following items are present in the figure legend, table legend, main text, or Methods section.

n/a	Confirmed	
☐	☒	The exact sample size (*n*) for each experimental group/condition, given as a discrete number and unit of measurement
☒	☐	A statement on whether measurements were taken from distinct samples or whether the same sample was measured repeatedly
☐	☒	The statistical test(s) used AND whether they are one- or two-sided<br>*Only common tests should be described solely by name; describe more complex techniques in the Methods section.*
☒	☐	A description of all covariates tested
☒	☐	A description of any assumptions or corrections, such as tests of normality and adjustment for multiple comparisons
☐	☒	A full description of the statistical parameters including central tendency (e.g. means) or other basic estimates (e.g. regression coefficient) AND variation (e.g. standard deviation) or associated estimates of uncertainty (e.g. confidence intervals)
☐	☒	For null hypothesis testing, the test statistic (e.g. *F*, *t*, *r*) with confidence intervals, effect sizes, degrees of freedom and *P* value noted<br>*Give P values as exact values whenever suitable.*
☒	☐	For Bayesian analysis, information on the choice of priors and Markov chain Monte Carlo settings
☒	☐	For hierarchical and complex designs, identification of the appropriate level for tests and full reporting of outcomes
☒	☐	Estimates of effect sizes (e.g. Cohen's *d*, Pearson's *r*), indicating how they were calculated

Our web collection on statistics for biologists contains articles on many of the points above.

Software and code

Policy information about availability of computer code

Data collection	NA
Data analysis	GRASS GIS 7.4, R, GDAL 2.0, QGIS 2.18

For manuscripts utilizing custom algorithms or software that are central to the research but not yet described in published literature, software must be made available to editors/reviewers. We strongly encourage code deposition in a community repository (e.g. GitHub). See the Nature Research guidelines for submitting code & software for further information.

Data

Policy information about availability of data

All manuscripts must include a data availability statement. This statement should provide the following information, where applicable:

- Accession codes, unique identifiers, or web links for publicly available datasets
- A list of figures that have associated raw data
- A description of any restrictions on data availability

CHIRPS rainfall data is freely available at the Climate Hazard Group (http://chg.geog.ucsb.edu/data/chirps/). SMOS and L-VOD data are available via CATDS (Centre Aval de Traitement des Données SMOS) at https://www.catds.fr/. GEOV2 data are kindly provided by the Copernicus Global Land Service (http://land.copernicus.eu/global/). VOD data was provided by Yi Liu (available at http://www.wenfo.org/wald/global-biomass/). The copyright for the field data remains at the CSE, Senegal. These data can only be redistributed upon request. Commercial very high resolution satellite images were acquired within the NextView license program. The copyright remains at DigitalGlobe and a redistribution is not possible.

Field-specific reporting

Please select the one below that is the best fit for your research. If you are not sure, read the appropriate sections before making your selection.

☐ Life sciences ☐ Behavioural & social sciences ☒ Ecological, evolutionary & environmental sciences

For a reference copy of the document with all sections, see nature.com/documents/nr-reporting-summary-flat.pdf

Ecological, evolutionary & environmental sciences study design

All studies must disclose on these points even when the disclosure is negative.

Study description	This study analyses 30 years of field and satellite data on herbaceous and woody foliage mass and rainfall. The impact of changing rainfall patterns on herbaceous and woody foliage production is studied.
Research sample	The field data are based on annual surveys at 9 sites, each a 1 km transect. The data were collected by our Senegalese colleagues at CSE following a protocol developed by Pierre Hiernaux in the 1980s. The data collection and methods are in detail described in https://www.mdpi.com/2072-4292/7/7/9122
Sampling strategy	Along the 1 km transect, 40 quadrants are evenly distributed, each 1 m2, and all herbaceous mass within the quadrants is harvested, dried and weighted. The woody foliage mass is calculated with allometric models, calibrated with fresh leave mass and based on tree measurements repeated every 2 years. See https://www.mdpi.com/2072-4292/7/7/9122 for details.
Data collection	The data were recorded by the CSE in Senegal, each year at the end of the dry season (end of September).
Timing and spatial scale	Start 1987, end 2016. No data was collected in 2004.
Data exclusions	No data were excluded.
Reproducibility	Satellite data were used to verify the patterns found in the field data.
Randomization	NA
Blinding	NA

Did the study involve field work? ☒ Yes ☐ No

Field work, collection and transport

Field conditions	Field work was conducted at the end of the rainy season. At this time of the year, the peak of the production is reached.
Location	The field sites are located in the Ferlo region of northern Senegal, a pastoral zone of the Sahel.
Access and import/export	The Senegalese authorities are involved in the data collection.
Disturbance	NA

Reporting for specific materials, systems and methods

We require information from authors about some types of materials, experimental systems and methods used in many studies. Here, indicate whether each material, system or method listed is relevant to your study. If you are not sure if a list item applies to your research, read the appropriate section before selecting a response.

Materials & experimental systems

n/a	Involved in the study	
☒	☐	Antibodies
☒	☐	Eukaryotic cell lines
☒	☐	Palaeontology
☒	☐	Animals and other organisms
☒	☐	Human research participants
☒	☐	Clinical data

Methods

n/a	Involved in the study	
☒	☐	ChIP-seq
☒	☐	Flow cytometry
☒	☐	MRI-based neuroimaging
