# Supplementary material for: Changes in rainfall distribution promote woody foliage production in the Sahel
Source: Commun Biol. 2019 Apr 23;2:133. doi: 10.1038/s42003-019-0383-9 (PMC6478729; doi:10.1038/s42003-019-0383-9)
Supplement: Supplementary file 2 — Supplementary information [file 42003_2019_383_MOESM2_ESM.pdf]

Supplementary information

Supplementary Figures

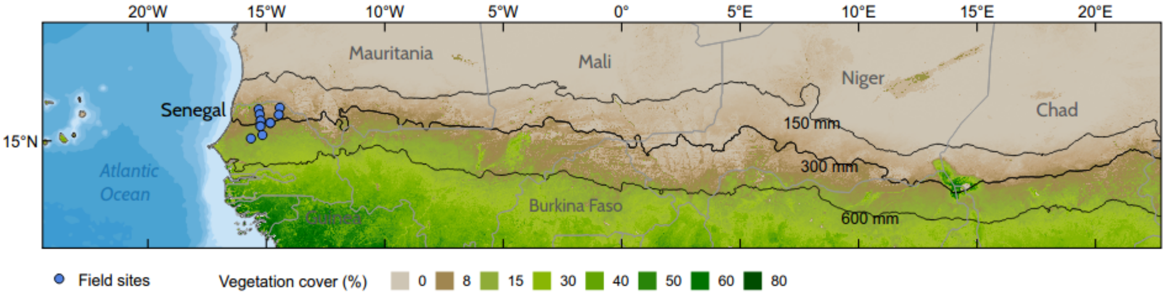

Supplementary Figure 1 | Study sites in the grazing lands of the sandy Ferlo at the most western Sahel. *Location of the sandy Ferlo and mean annual vegetation cover (GEOV2 FCover) in percent. Isohyets derived from average annual rainfall (CHIRPS v2 1982–2016) mark 150, 300 and 600 mm.*

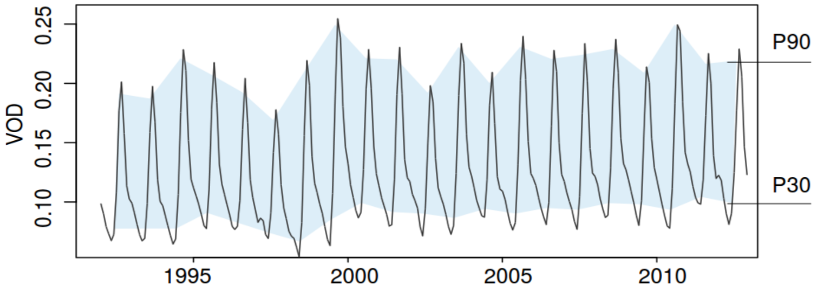

Supplementary Figure 2 | Monthly VOD is averaged for Western Sahel from 1992 to 2012. *The upper bound of the blue area is the 90th percentile used as proxy for total green vegetation mass and the lower bound is the 30th percentile used as proxy for woody plant foliage (WPF). Aboveground herbaceous mass (AGH) is estimated by subtracting WPF from total green vegetation mass (AGH = p90–p30).*

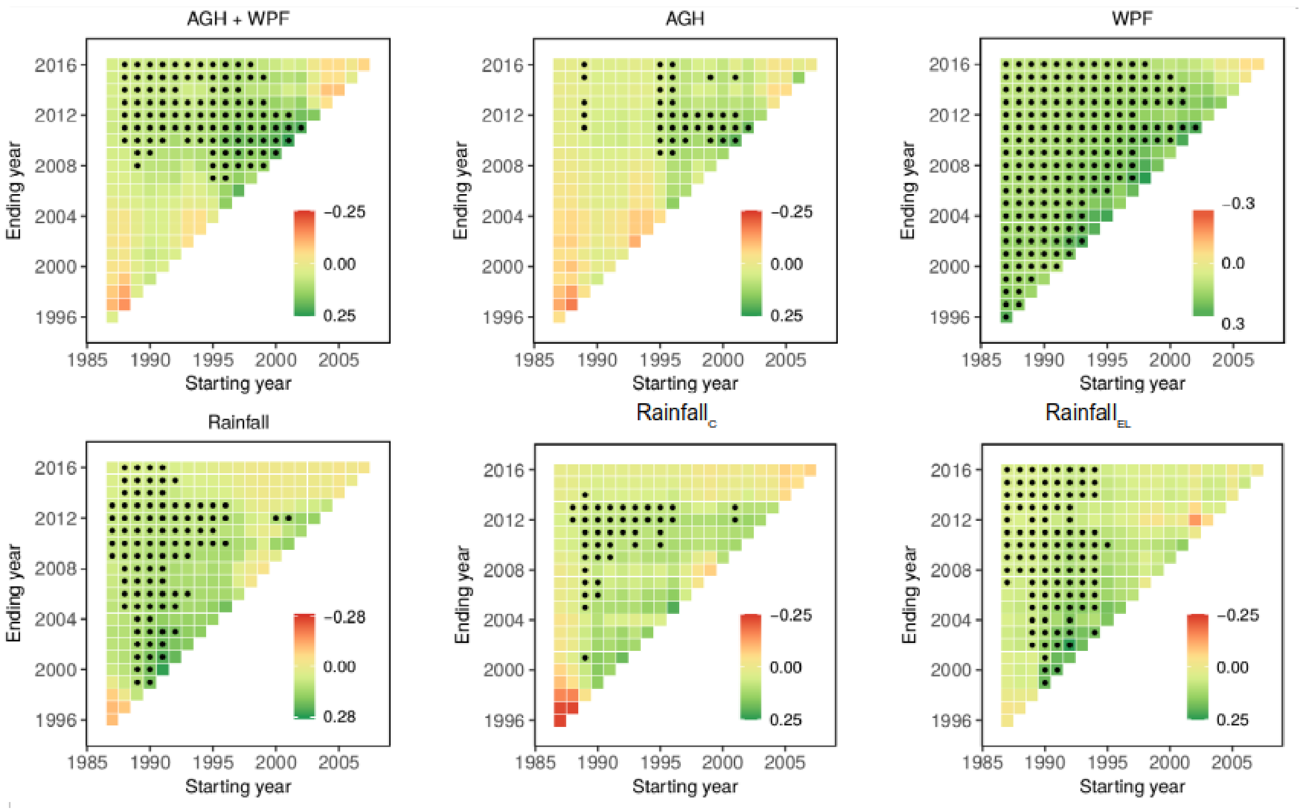

Supplementary Figure 3 | Sensitivity of the trends to the period of analysis. Points mark significant ($P < 0.05$) trends*. Total vegetation mass (AGH+WPF), AGH, WPF, as well as annual rainfall, core wet season rainfall (rainfall$_C$) and early/late rainfall (rainfall$_{EL}$) are shown. AGH = aboveground herbaceous mass, WPF = woody plant foliage.*

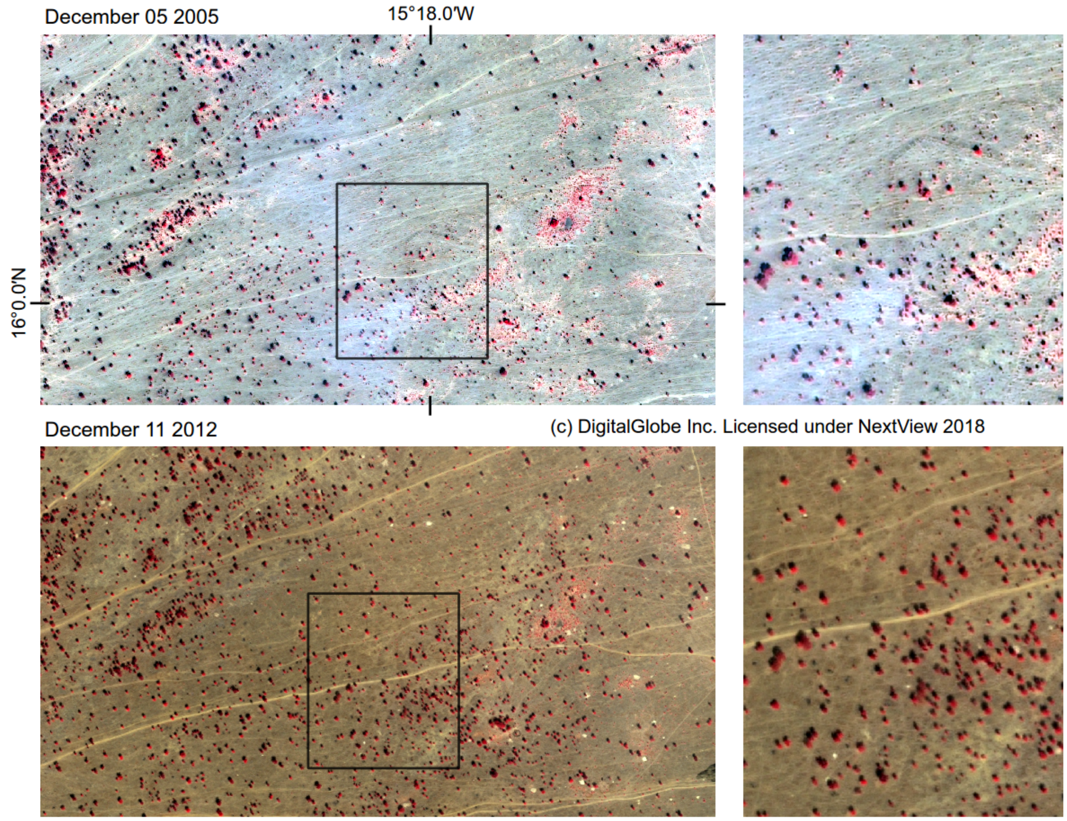

Supplementary Figure 4 | Woody vegetation in the northern Ferlo north of the deep well Widou Thiengoly. *The scene from 2005 is a Quickbird-2 image and the scene from 2012 is from the WorldView-2 satellite. Measured woody cover at the field site close by was 4.89% in 2007 and 7.24% in 2013. Woody plant foliage mass (WPF) increased in this area for the entire period (1987–2016) with +21 kg ha*$^{-1}$ *yr*$^{-1}$*. Only few image pairs were available, cloud cover was present in most images, and the period between the pairs differs, so no systematic analysis on very high spatial resolution imagery was possible.*

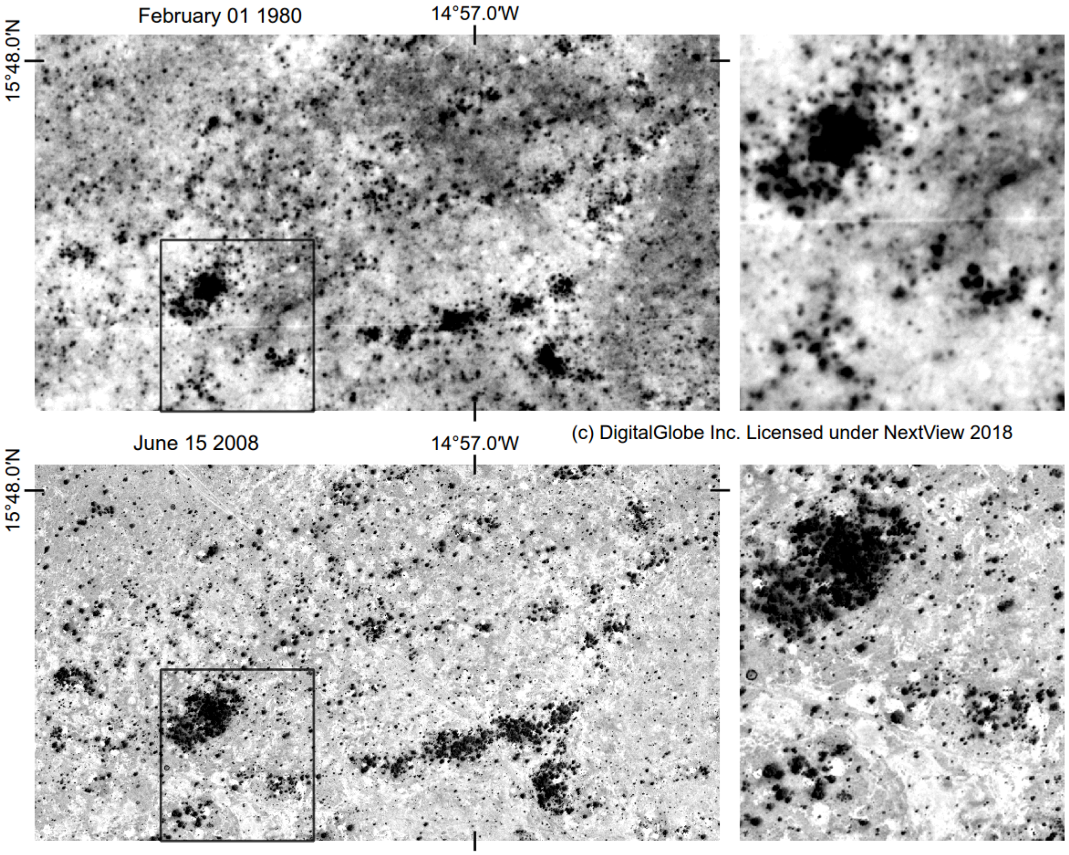

Supplementary Figure 5 | The woody vegetation between 1980 to 2008. *Although the quality of the aerial photo from 1980 cannot be compared with the panchromatic band of Worldview-1 (2008), the image pair shows no considerable change in woody cover over the 2 decades, and even many larger trees remain unchanged. Although most of the study area is covered by these image pairs (1980–2008), the poor quality of the aerial photos impedes a systematic analysis. Moreover, the timing of the aerial photos (1980) was before the major drought in 1984, and our field data collection starts in 1987. Even though a direct and systematic comparison was not possible, a careful visual screening showed that the woody vegetation over this period was rather stable. In total, we studied 30 image pairs, each about 150 km². The chosen examples reflect the results.*

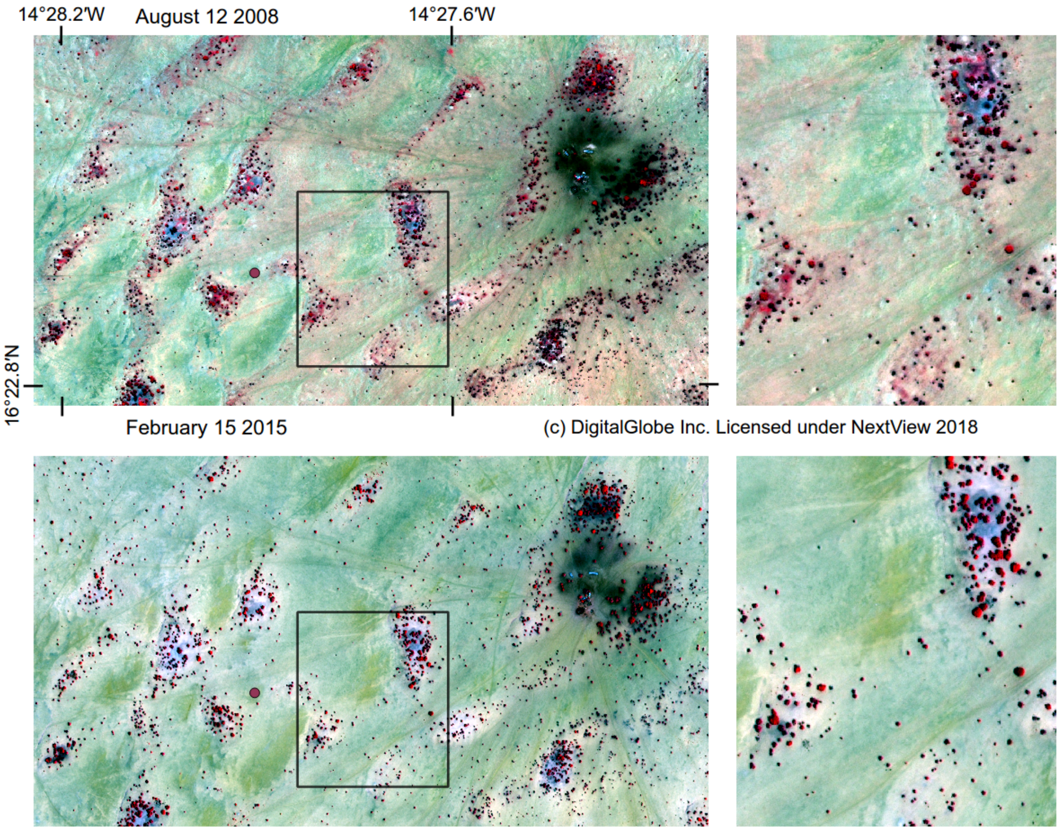

Supplementary Figure 6 | Woody vegetation of the eastern Ferlo 2008 and 2015. *Woody cover measured was 4.77% in 2009 and 5% in 2015. Woody plant foliage mass (WPF) at this site increased by +8 kg ha^{-1} yr^{-1} over the full period (1987–2016).*

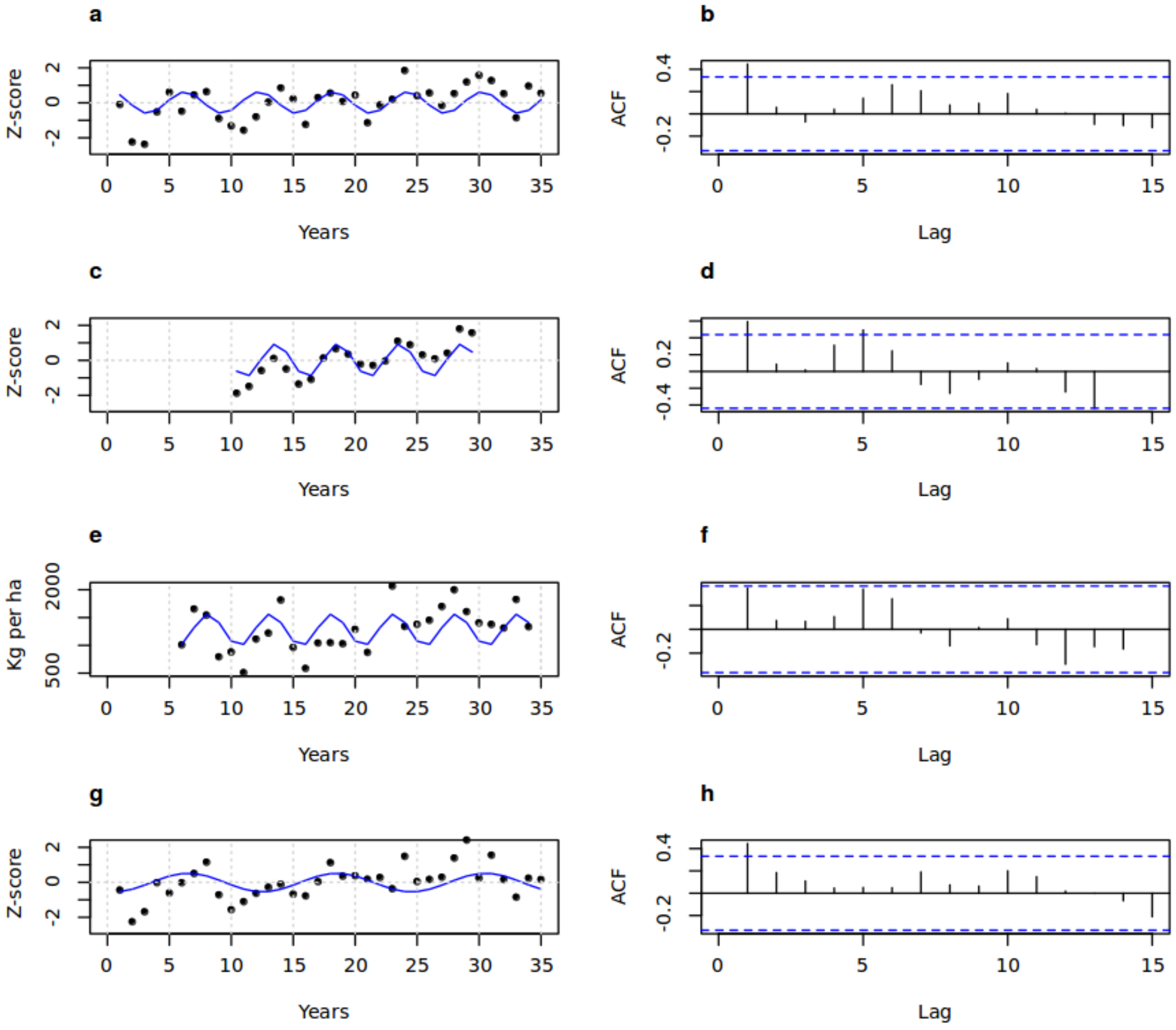

Supplementary Figure 7 | Cyclic patterns in vegetation dynamics*. Annual vegetation production follows a periodic pattern shown as sinusoidal term (1st harmonic from a Fourier transformation (left column) and autocorrelation lag (right column). (a+b) GIMMS3g P90 1982–2016. (c+d) VOD P90 1992–2011. (e+f) Field data on green vegetation mass 1987–2016. (g+h) CHIRPS annual rainfall 1982–2016.*

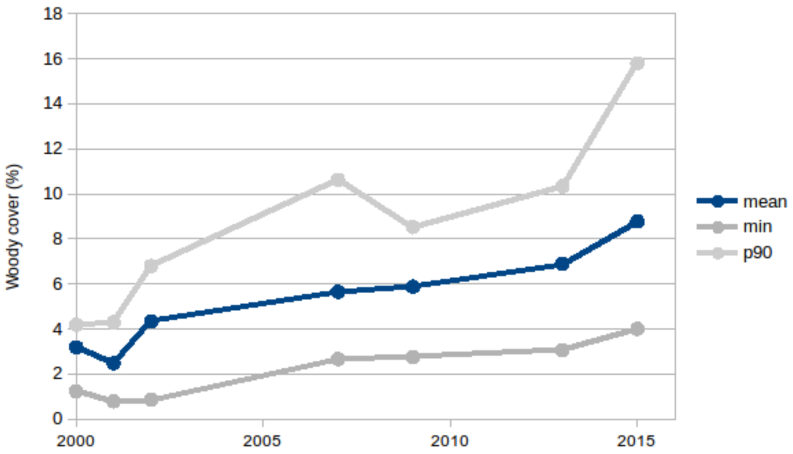

Supplementary Figure 8 | Field measured woody cover. *Measured approximately every 2 years at the same sites as AGH and WPF, but starting only in 2000. Here we show the min, mean and 90th percentile of the 9 field sites.*

Supplementary Tables

Supplementary Table 1 *| All data sets used in the analysis. AGH = aboveground herbaceous mass, WPF = woody plant foliage. FCover = vegetation cover. VOD = vegetation optical depth. NDVI = normalized difference vegetation index.*

**Data**	**Period**	**Spatial resolution**	**Parameter**
Field data	1987–2016	9 sites, each 1 km	AGH, WPF
GEOV2	1999–2016	1 km	FCover
MODIS	2000–2016	5.6 km	NDVI
GIMMS3g	1982–2016	8 km	NDVI
VOD	1992–2012	25 km	VOD
SMOS-IC	2010–2016	25 km	L-VOD
CHIRPS v2	1982–2016	5.6 km	Daily rainfall
