# Supplementary material for: Changes in rainfall distribution promote woody foliage production in the Sahel
Source: Commun Biol. 2019 Apr 23;2:133. doi: 10.1038/s42003-019-0383-9 (PMC6478729; doi:10.1038/s42003-019-0383-9)
Supplement: Supplementary file 3 — Description of additional supplementary items [file 42003_2019_383_MOESM3_ESM.docx]

Description of additional supplementary items

**Supplementary Information.docx**: File containing Supplementary Figs 1-8 and Supplementary Table 1

**Supplementary Data.csv**: File containing the data used in this study as textfile

Abbreviations:

AGH+ẄPF: total green vegetation production

RainfallC: core wet season rainfall

RainfallEL: early and late rainfall

SMOSp90: 90^th^ percentile of SMOS L-VOD

VODp90: 90^th^ percentile for VOD

AGH: aboveground herbaceous mass

WPF: woody plant foliage

AGHsd: standard deviation of AGH

WPFsd: standard deviation of WPF

FCOVERp30: 30^th^ percentile of GEOV2 FCOVER

FCOVERp90: 90^h^ percentile of GEOV2 FCOVER

MODp30 and p90: MODIS percentiles

GIMMS p30 and p90: percentiles from GIMMS 3g v1

p90p30: difference between 90^th^ and 30^th^ percentiles

VOD, MOD, FC, SMOS AGH and WPF: AGH and WPF estimated with VOD, MODIS, GEOV2 FCOVER and SMOS by using a linear regression with the field data

WPF, AGH, total Sahel: vegetation mass for Sahel estimated with VOD

Sahelp90: 90^th^ percentile of VOD for Sahelp90

Sahelp90p30: difference between 90^th^ and 30^th^ percentile from VOD for Sahelp90

Sahel rainfallC: core wet season rainfall for Sahel

Sahel rainfallEL: early and late rains for Sahel
